# Nursing consultation in childcare in nurses' and managers' voices: a mixed methods study

**DOI:** 10.1590/0034-7167-2024-0657

**Published:** 2025-12-08

**Authors:** Amanda Teixeira Martins, Vanessa Faria de Freitas, Larissa Carvalho de Castro, Thayane Ingrid Xavier de Andrade, Wiara Viana Ferreira, Elaine Cristina Rodrigues Gesteira

**Affiliations:** I Universidade Federal de São João del-Rei. Divinópolis, Minas Gerais, Brazil; II Universidade Estadual de Campinas. Campinas, São Paulo, Brazil

**Keywords:** Child Health, Primary Health Care, Comprehensive Health Care, Growth and Development, Child Care, Salud Infantil, Atenação Primaria de Salud, Atenação Integral de Salud, Crecimiento y Desarrollo, Cuidado del Niño

## Abstract

**Objectives:**

to investigate the work process of nurses in relation to nursing consultations in childcare.

**Methods:**

exploratory research with a mixed approach, in which quantitative data were collected concomitantly with qualitative data. Nurses and managers were included in the research.

**Results:**

interviews were conducted with nine nurses and eight managers of Family Health Strategy units in the Central-West region of Minas Gerais. After analysis, four thematic categories emerged: Most common guidelines during childcare nursing consultations; Advances in childcare nursing consultations; Challenges in childcare nursing consultations; and Nurses' role in childcare consultations.

**Final Considerations:**

nurses are the professional interlocutors of actions involving child care and monitoring in the Health Care Network, but there are challenges that need to be overcome in order to improve coverage in the care of these children and their families, aiming for comprehensiveness and longitudinality.

## RESUMEN

**Objetivos:**

investigar el proceso de trabajo de las enfermeras en relação a las consultas de enfermería en el cuidado infantil.

**Métodos:**

investigação exploratoria con enfoque mixto, en la que se recolectaron datos cuantitativos concomitantemente con datos cualitativos. Enfermeras y gerentes fueron incluidos en la investigação.

**Resultados:**

se realizaron entrevistas con nueve enfermeros y ocho gestores de unidades de la Estrategia de Salud de la Familia de la região Centro-Oeste de Minas Gerais. Luego del análisis surgieron cuatro categorías temáticas: Pautas más comunes durante las consultas de enfermería de puericultura; Avances en la consulta de enfermería en el cuidado infantil; Desafíos de la consulta de enfermería en el cuidado infantil; y el papel de la enfermera en las consultas de puericultura.

**Consideraciones Finales:**

la enfermera es el interlocutor profesional de las acciones que involucran el cuidado y seguimiento del niño en la Red de Atenação a la Salud, pero existen desafíos que necesitan ser superados para mejorar la cobertura en la atenação a estos niños y sus familias, buscando la integralidad y longitudinalidad.

## INTRODUCTION

Early childhood, the period from 0 to 5 years of age, is a decisive moment for the healthy development of human beings. Since the 1980s, with the Comprehensive Child Healthcare Program, there has been a challenge to establish and implement policies and programs that guarantee ideal conditions for full child growth and development with a focus on comprehensive care^(1)^.

According to the Brazilian National Policy for Comprehensive Child Healthcare, strategic actions for universal and equitable care include: updated access to the Child Health Booklet; training of healthcare professionals to care for children and their families in Primary Healthcare (PHC); social mobilization and a committee of experts for the Comprehensive Development of Early Childhood in the Brazilian Health System; and support in the implementation of the Brazilian National Plan for Early Childhood^(1)^.

In this context, monitoring of child growth and development must be carried out in PHC through the Family Health Strategy (FHS), and nurses are responsible for carrying out nursing consultations in childcare, establishing a link between the service and the community, providing communication and a harmonious relationship with the child and their family, in addition to systematizing care, in order to identify health problems and establish a plan with the family for decision-making, directing them to care through injury promotion, prevention, and rehabilitation^(2-4)^.

Therefore, childcare should be shared between physicians and nurses, in order to ensure the child's follow-up, as recommended by the Ministry of Health, with seven consultations in the first year of life, two in the second, and one annual consultation, from 2 years of age until the child is 9 years old^(5)^. In addition to monitoring child growth and development, nursing consultations are an opportune time to create strategies that place family members as active participants in child care, including the exercise of parenting, relationships with the internal and external environment, as well as stimulus for harmonious growth and development, which will determine adulthood, being the foundation for the future with healthy, humanitarian individuals who are capable of relating and producing, in order to build a favorable environment for future generations^(6)^.

However, some studies have highlighted weaknesses in childcare nursing consultations in different states of the country, such as: incomplete completion of nursing consultations in terms of anamnesis, physical examination, health education and welcoming families with their unique characteristics^(7,8)^; lack of training for nurses, consultations focused on the disease rather than prevention, guidelines restricted to breastfeeding and hygiene^(5)^; and also barriers inherent to the health unit itself, such as the shortage of human and material resources, among others that can hinder the family and child's access to effective childcare^(9)^.

Considering the above, the question is: how have nurses conducted nursing consultations in childcare? What are the facilities and difficulties encountered by nurses and managers in carrying out childcare?.

It is believed that, by investigating nursing consultations in childcare, the nursing process performed will be identified and the human and physical barriers that impede effectiveness in the follow-up for comprehensive care of children will be identified, in order to contribute to proposals that aim at the quality of care provided.

## OBJECTIVES

To investigate nurses' work process in relation to nursing consultations in childcare.

## METHODS

### Ethical aspects

The ethical and legal precepts regarding research involving human beings were met in accordance with Resolutions 466/2012 and 510/2016. The article was approved by the *Universidade Federal de São João del-Rei* Research Ethics Committee, *Campus Centro-Oeste Dona Lindu*, and Certificate of Presentation and Ethical Consideration. All participants signed the Informed Consent Form.

### Study design

This is mixed methods research, in which quantitative data (QUAN) were collected concomitantly with qualitative (Qual) data^(10)^. The QUAN study was exploratory, and the Qual part was descriptive, anchored in hermeneutic phenomenology, in which the understanding of the lived experience is given by the interpretation of researchers^(11)^, but both data had the same weight value. The mixed approach was guided by the Mixed Methods Appraisal Tool, which was developed to critically assess different study designs, identifying relevant methodological criteria to assess the quality of studies, such as mixed methods studies^(12)^.

This mixed approach was chosen because it allows for a comprehensive and in-depth response to the phenomenon studied, considering the guiding axes that involve nursing consultation in childcare from a quantitative perspective, and with Qual data, there was an opportunity to understand the experience lived. The use of a single method would imply knowledge of the phenomenon studied, only in a quantitative manner, about what nurses knew about childcare, while conducting in-depth interviews offered the opportunity to clarify to the researchers the actions carried out during consultation and to understand it from the perspective of nurses and managers of the selected health districts.

In this way, the mixed method allowed the convergence of data, supporting interpretation of results, which led to knowledge and understanding about the perceptions and attitudes that involve childcare practice carried out by nurses in the multidisciplinary team, aiming to highlight the advances achieved in this practice in child healthcare and the challenges that still exist.

### Study setting

The study was carried out in a municipality in the Central-West region of Minas Gerais, which has eight health districts: Central, North, Northeast, Southeast, South, Southwest, West, and Northwest.

Thus, nurses and managers of a FHS from each health region, eligible by convenience, were included in the study, which were fields of practice of undergraduate and residency *lato sensu* graduate courses of a public university in the Central-West region of Minas Gerais. Prior contact was made with nurses and managers, by means of a telephone call, at the respective health units. Professionals who were away due to medical leave, unpaid leave or vacation were excluded.

### Data collection and organization

Data were collected from September 2023 to June 2024. The sample consisted of nine nurses and eight managers. Data collection with the nurses began through an open interview based on questions about anamnesis, physical examination, health education, conduct at the end of consultations, and facilities/challenges, in order to know and understand their perceptions about the consultations performed. It should be noted that the interviews were conducted in private locations in the health unit and after participants' consent.

At the end of each interview with a nurse, a questionnaire was applied containing ten multiple-choice questions addressing specific points of the nursing consultation according to the Ministry of Health's Child Health Booklet, in order to investigate the work process^(13)^. However, the questions were different from those asked in the interview, in order to not influence the answers and to complement the study.

For managers, an open interview was applied with two central questions involving their vision in relation to the consultation carried out by nurses, and the challenges and potential existing in the area covered by their management.

In the collection stage (Qual), the interviews were recorded and transcribed after completion. Participants were identified with the letters "N" (nurse) and "M" (manager), following the numerical sequence of their interview (N1, N2, N3, M1, M2, M3, successively). The data from the interviews went through three phases recommended by content analysis^(14)^.

The three stages were pre-analysis, material exploration, coding and classification, and treatment of results obtained and interpretation, with the elaboration of thematic categories. The codes generated were analyzed by three researchers simultaneously and independently.

It should be noted that, to conduct the interviews and apply the questionnaires, two researchers of this study were the executors, who made telephone contact at the health center of each selected district, making the invitation and scheduling the day and time preferred by the nurse and the respective manager.

The data collection team consisted of a nurse researcher, who holds a master's and a doctoral degree in nursing, and other researchers who are nursing students, one of whom is a scientific initiation scholarship holder. All of them had experience in conducting interviews, in addition to experience in providing nursing consultations in childcare. Although the FHS units were fields of undergraduate and graduate practice, professionals who were not known to the researchers were sought out for convenience in order to ensure methodological rigor.

### Data analysis

To analyze the QUAN data, the data were entered and stored in Microsoft Office Excel. They were then analyzed in percentages, according to the answers given to the questions, which were described in a chart indicating the alternative that would be correct, the most frequent answer given by the nurses and the percentage of correct answers for each question asked.

It should be noted that no comparison or association tests were applied to the questionnaire results, as the sample was limiting for making statistical inferences; however, the results of descriptive statistics are presented. The final version of the database was checked by two researchers.

Data triangulation was performed using the empirical material from questionnaires and interviews, in order to respond to the objective through complementary information, which allowed a broader view of the nursing process carried out in childcare.

## RESULTS

The study data were extracted simultaneously through interviews and questionnaires administered to nurses. The interviews were applied first to avoid bias, since the questionnaire contained questions that could influence the responses. However, analysis was triangulated, allowing four thematic categories to be identified: Most common guidelines during childcare nursing consultations; Advances in childcare nursing consultations; Challenges in childcare nursing consultations; and Nurses' leading role in childcare consultations.

The most common conduct during childcare was mainly related to exclusive breastfeeding/nutrition (100% correct answers in the questionnaire), supplementation and other medications in use (66.6% correct answers), guidelines for accident prevention, stimulation of child development (33.3%) and guidelines for completing the vaccination schedule. Moreover, it was also observed that some professionals schedule the next childcare appointments.

The following facilities stood out: formation of a bond with the family from prenatal care onwards; child assessment instruments; promotion of health education during consultations; improvement of health indicators; possibilities of monitoring with specialized service programs; technological advances; scheduled care schedule; and actions and programs aimed at primary care.

Regarding the challenges, the following stood out: the need for greater adherence by parents and guardians to childcare consultations, weaknesses in the integration of the multidisciplinary team, the need for professional updating, the high demand for nurses in the healthcare service and the location of the unit, which makes user access difficult and limitations in integration with other care sectors.

Furthermore, the managers signaled the importance of nurses' role as an essential member of the multidisciplinary team, holding obligations and commitments related to child health promotion, prevention and maintenance.

Nurses' and managers' speeches were analyzed and presented according to the codes and categories presented in Chart 1.

**Chart 1:** Categories, codes and discourse of the interviewees, Divinópolis, Minas Gerais, Brazil, 2023.

Code	Category	Nurses' and managers' speeches
Breastfeeding Complementary feeding Prophylactic medications Information on preventing childhood accidents Stimulation for child development Identification of delays Vaccinations Scheduling with other professionals	1 - Most common guidelines during childcare nursing consultations	*I guide parents on nutrition, breastfeeding, exclusive breastfeeding up to 6 months. Breastfeeding up to 2 years of age. Introduction to healthy eating [...]. N2*[...] *I provide guidance on care, importance, benefits of breastfeeding, the various foods, benefits of exclusive breastfeeding for both the child and the mother, as well as financially, as formula milk is quite expensive* [...]. *So, always be careful with feeding natural foods, to continue breastfeeding, even with the introduction of solid foods* [...]. N3 *If she is exclusively breastfed, I will encourage breastfeeding; I will congratulate the mother for exclusive breastfeeding; I will give general guidance on child care and when it is necessary to use some type of vitamin supplement* [...]. N7 [...] *I talk a lot about the medication, which is different, especially ferrous sulfate, and that from 6 months onwards, we advise on the importance of the medication, as well as vitamin D* [...]. N8 [...] *accident prevention, in this case, the sleeping position, preferably sleeping in the crib* [...]. *When she starts walking, crawling, be careful with toy parts, small pen caps, coins, small parts, staying at home, furniture corners, knife drawers, the sharp utensils drawer is taken, these things, basically.* N3 [...] *If I have any movement delay, I can ask the physiotherapist at the unit for guidance. She helps a lot with this issue. Depending on my age, I can walk to PIPA* [...]. N1 [...] *stimulation guide, I teach the exercises that the mother has to do at each stage"¦ to stimulate the baby's development* [...]. N5 *I show all the development and growth charts. I usually guide how to stimulate the baby according to age* [...]. N9 [...] *I schedule a return visit or referral to a point in the network or to another professional when necessary.* N4
Family bond Role of the nurse Improvement in health indicators Access to care technologies Quality monitoring Shared childcare	2 - Advances in childcare nursing consultations	[...] *create a bond with the family, and this bond is actually created from prenatal care, because as a nurse, I do prenatal consultations. So, this bond is important, because then we will have a longitudinality of the user* [...]. N1 [...] *family involvement is a huge facilitator*. N2 *Sometimes, the pediatrician focuses more on the clinical issue and forgets to give other guidance. So, there are many mothers who seek childcare at the health unit because sometimes we* [referring to the nursing professional] *talk a little more with her, right, we give more guidance* [...]. N7 *Improvement of indicators, when we think about vaccination issues, SISVAN issues, which is nutritional surveillance, but what I see most is this issue itself, mainly development. When we, the nurse, detect any alteration, it is already referred to the Early Intervention Program. We are providing qualified assistance, when it comes and mainly detecting it as early as possible*. M1 *Advances in technology make it easier for nurses to research, stay up to date, and talk to mothers. Applications that can be used to monitor the child's development to know whether or not it is appropriate for their age* [...]. M2 *But one advancement that I also see is in the issue of agendas, having agendas reserved for childcare*. M5 *I think that the main advance is this, that we are able to monitor almost 100% of these children. It is rare that there is a month in which we are not able to monitor 100% of children. Everyone already knows about the quality of this monitoring in the unit, so this was our biggest advance, our biggest gain to date* [...]. M7 [...] *the programs, in a way, are already here to be able to overcome these difficulties that we would have as a base, which are medications. We have a lot of access to vitamins here that we didn't have before, right? I think that what has advanced the most is this view of childcare. It is part of all specialties, it doesn't belong only to nurses, and they don't have to do these consultations alone* [...]. M8
Articulation of the Healthcare Network Shortage of professionals Lack of knowledge about child health Lack of training Nursing overload Lack of parental involvement Difficulties in accessing the unit (geographical location) Difficulties in referring the child to other Healthcare Network services	3 - Challenges in childcare nursing consultations	[...] *need for a more structured multidisciplinary team to help us with the monitoring, because, many times, we feel very alone, right? Sometimes there is a professional, but they do not come to the unit in sufficient numbers. Sometimes, it is once a week, only half a day. They do not meet, we cannot discuss the cases properly, it is super fragmented* [...]. N4 [...] *of difficulty, I think that when we don't have knowledge, the lack of knowledge about something that we have, right?!* [...] *but here there is no training, no* [...] *I don't even know when was the last time we had some permanent education in child health* [...]. *Nowadays, there is only one nurse for everything in the unit, for everything, everything, everything, for all these schedules, all these spontaneous demands, always being available for this, makes it difficult for us to monitor the way we need to, you know?* N5 *I think that parents need to be more aware of the importance of childcare monitoring. Sometimes, I see that many people don't understand or don't want to follow the instructions given by nurses or other healthcare professionals. And I think that it's harder to follow some instructions in less privileged areas* [...]. M1 [...] *I also challenge the nursing professional's schedule in the health unit, given all the other functions they have. It is difficult for them to set aside a schedule for childcare* [...] *the first thing they do is cancel the childcare schedule, because it ends up not being considered very urgent, and this ends up discrediting the service. Parents will not take it very seriously.* M2 *Here, the main challenge we have is a question of logistics* [...] *this part of the community that lives far away, they don't have direct buses to here. So, our main challenge is not technical, it's not about monitoring, it's this question of the logistics of locating the unit* [...]. M7 [...] *what I have a lot of reservations about is that when we need a third sector, a second sector, that's when we start to encounter challenges, if we find a child who has some limitations* [...] *to be able to guide this child to more specialized care* [...] *we as a unit, we encounter some obstacles* [...]. M8
Recognition of the role played by nurses Challenges for families in caring for children Valuing nursing consultations	4 - Nurses' leading role in childcare consultations	[...] *the nurse is a professional trained and qualified to perform such a procedure* [...]. M1 *The nurse is a staff member who is closest to the unit. They are there every day. It is easier for them to monitor the child, so it is important for nurses to monitor the child according to the recommended dates in order to diagnose any delays and be able to intervene at the right time, and to provide guidance to the child's parents or guardians in the correct way* [...] *they are the person who are closest to the user.* M2 [...] *I see that it is a moment in which the family often has even greater support than perhaps a pediatric consultation* [...]. M3 *If we were to centralize childcare, let's say, in the physician's office or even in a pediatrician's office, we wouldn't be able to meet the demand, there would be a very outdated demand for care* [...]. M5 [...] *nurse on the front line, right? She is the one who will really follow this development that I think is a priority, even this nursing care* [...] M6 [...] *they have very good, advanced technical knowledge, it is an enormous technical capacity to be able to accompany these children with childcare and in addition to having the ability to approach those responsible, the parents, in relation to this* [...] M7.

The questionnaire was applied to the nurses (nine) included in the study. Among the topics that make up childcare, Chart 2 shows the questions asked and the identification of hits and errors in the questionnaire.

**Chart 2:** List of questions in the questionnaire, correct alternative and answer that participants selected most often and percentage of correct answers for each question (n=nine participants), Divinópolis, Minas Gerais, Brazil, 2023.

Question 01) Select the alternative that clearly corresponds to the definition of a nursing consultation in childcare.		
Correct alternative	Most frequent answer	Correct answer (%)
e) All alternatives are correct.	e) All alternatives are correct.	88.90%
Question 02) Nursing consultation in childcare is carried out up to the age of:		
Correct alternative	Most frequent answer	Correct answer (%)
b) Up to 9 years, 11 months and 29 days	b) Up to 9 years, 11 months and 29 days	44.40%
Question 03) You are carrying out a nursing consultation in childcare for a premature newborn. Considering that the newborn was born two months ago, at 29 weeks, select the correct alternative for the corrected age:		
Correct alternative	Most frequent answer	Correct answer (%)
c) 37 weeks	c) 37 weeks	77.70%
Question 04) Regarding vitamin A supplementation, it is incorrect to state that:		
Correct alternative	Most frequent answer	Correct answer (%)
b) Children aged 1 to 3 years should be supplemented	b) Children aged 1 to 3 years should be supplemented	44.40%
Question 05) Regarding the administration of prophylactic ferrous sulfate supplementation, it is correct to state:		
Correct alternative	Most frequent answer	Correct answer (%)
e) All alternatives are correct	a) For full-term newborns, with appropriate weight for gestational age and breastfed, the recommendation is 1 mg/kg weight/day from the 6th month to the 24^th^ month of life.	22.20%
Question 06) Regarding complementary feeding, it is correct to state that:		
Correct alternative	Most frequent answer	Correct answer (%)
b) Children who are exclusively breastfed, from 6 months onwards, should start complementary feeding and drinking water.	b) Children who are exclusively breastfed, from 6 months onwards, should start complementary feeding and drinking water.	100%
Question 07) Which developmental milestone is not present at 2 months?		
Correct alternative	Most frequent answer	Correct answer (%)
a) Active search for objects	a) Active search for objects	33.30%
	b) Holds objects	
Question 08) Until what age should you check the head and abdominal perimeter?		
Correct alternative	Most frequent answer	Correct answer (%)
c) Up to 2 years of age	c) Up to 2 years of age	66.70%
Question 09) What is the recommended age to start taking diapers off a child?		
Correct alternative	Most frequent answer	Correct answer (%)
c) Between 2 and 3 years	d) None of the alternatives are correct.	22.20%
Question 10) How many childcare appointments should a child have before the age of 2?		
Correct alternative	Most frequent answer	Correct answer (%)
c) Nine consultations	c) Nine consultations	66.70%

In items such as consultation definition and objectives, corrected age, and complementary feeding, more than 70% of correct answers were achieved, followed by physical examination, with verification of the age limit for measuring head and abdominal perimeter (66.7%), and the number of consultations that a child should have until 2 years of age (66.7%). On the other hand, the questions that obtained the lowest correct answers were related to vitamin A supplementation (44.4%), up to what age childcare should be performed (44.4%), developmental milestones (33.3%), prophylactic supplementation of ferrous sulfate (22.2%) and diaper removal (22.2%).

## DISCUSSION

As for the most common guidelines given by professionals, the most notable quantitative measure is the encouragement of breastfeeding, an action that supports the results obtained by the Ministry of Health's Brazilian National Study of Infant Feeding and Nutrition, which shows an increase in breastfeeding rates. When comparing the data with previous national surveys, based on breastfeeding indicators proposed by the World Health Organization, all indicators improved in Brazil. There was a 15-fold increase in the prevalence of exclusive breastfeeding among children under 4 months and 8.6-fold among children under 6 months^(15)^.

However, it is important to emphasize that several factors can influence the success of breastfeeding; among them, some are related to the mother, such as the stance and attitudes adopted in the breastfeeding situation, and others refer to sociocultural, demographic, socioeconomic and pre- and post-natal care factors^(16,17)^.

From this perspective, healthcare professionals' work is of great value in correctly initiating breastfeeding, supporting and encouraging mothers to overcome the likely early problems that recur in this process through simple but up-to-date guidance, transmitted with sympathy, ethics, patience and humanization, to assure and encourage the mother of her ability to breastfeed, if that is her wish^(15)^.

Furthermore, exclusive breastfeeding until the 6^th^ month, offered until 2 years of age or more, as well as a diverse diet, are determining factors for the provision of different nutrients, contributing to the prevention of nutritional deficiencies.

Other widely discussed guidelines are the stimulation of child development and monitoring of developmental milestones. It is known that child growth and development analysis should be done regularly so that early detection of changes is possible, enabling appropriate actions to be taken in a timely manner, with the aim of providing the child with opportunities for adequate development throughout childhood, contributing to their potential being manifested in a way that reflects positively throughout their lives^(18)^.

As for micronutrient supplementation, nurses' knowledge of ferrous sulfate prevails over other micronutrients. It can be stated that 77.8% of respondents know about the correct indication for full-term newborns, with appropriate weight for gestational age and breastfeeding, with iron supplementation at 1 mg/kg weight/day from the 6^th^ month to the 24^th^ month of life^(19)^.

As for vitamin A, 44.4% of respondents stated that children aged 1 to 3 years old do not need to be supplemented; this measure was justified by the low incidence of vitamin A-related hypovitaminosis in southeastern Brazil. On the other hand, between 2015 and 2022, 169 hospitalizations due to vitamin A deficiency were recorded in Brazil, a public health concern in the Northeast region for decades, especially in the semi-arid regions of Bahia, Rio Grande do Norte, Ceará and Recife, due to the high prevalence of clinical deficiency in several groups of children^(20)^.

In relation to vitamin D, only one of the nurses mentioned supplementation of this vitamin. Vitamin deficiency in children can result in several complications, such as delayed growth, bone deformities and fragility, and an increased risk of fractures. In addition, vitamin D also plays a vital role in regulating the immune system, and its deficiency can contribute to a greater susceptibility to respiratory infections and other diseases. Studies have shown associations between vitamin D deficiency in childhood and the development of chronic diseases in adulthood, such as type 1 diabetes and autoimmune diseases^(21)^. This fact reinforces the relevance of nursing counseling and interventions related to nutrition in childcare consultations.

It is important to emphasize that breastfeeding for up to 2 years or more and, consequently, the mother's diet are determining factors for the concentration of vitamins in children. Therefore, it is recommended that breastfeeding women consume a healthy diet daily with adequate levels to meet their daily needs^(2)^.

Regarding potty training, only 22.2% of participants knew the recommended age to start potty training, that is, between 2 and 3 years old^(13)^. Therefore, it is appropriate that professional qualifications are connected with the community's desires and needs^(22)^. Furthermore, it is essential that nurses guide parents to perceive the signs of children's neural maturity and carry out the potty training process with great understanding and naturalness^(13)^.

Regarding accident prevention, only one nurse discussed the subject when giving guidance to caregivers, although, according to the Brazilian Society of Pediatrics, accidents are the main cause of death among children aged 1 to 14 in the country. Around 90% of these accidents can be avoided with simple prevention measures^(23)^. Numerous accidents happen at home, where, in principle, children should have the freedom to move around, explore and develop in a healthy and safe way^(24)^. Given the above, it is essential that the subject is widely discussed during childcare consultations and that those responsible are warned about dangerous situations related to changes in children's cognitive and locomotive capacity during their development.

Similarly, only one of the nurses interviewed mentioned guidance on vaccination. According to the United Nations Children's Fund, the low vaccination coverage in the country leaves the child population exposed to diseases that were no longer a concern before, such as measles, which was eradicated in the country in 2016 and, in 2018, returned to the list of diseases in Brazil. In addition to measles, other diseases that are at risk of affecting children again are polio, meningitis, rubella and diphtheria^(25)^.

In this context, there is a clear need for primary care to strengthen nurses' training for childcare consultations, in addition to organizing actions for continuity of care and monitoring not only to meet spontaneous demand. To this end, it is necessary to actively seek out families from prenatal care onwards and integrate care with prevention and health promotion actions^(26)^.

In accordance with the data presented by nurses and managers, it is possible to note numerous advances in nursing consultation in childcare^(3)^. The formation of a bond with the family is highlighted as one of the advances highlighted in the interviews, important for the longitudinality of care, which is strengthened through a relationship of trust and respect, allowing for comprehensive care and long-term monitoring of these users^(27)^.

Furthermore, the implementation of actions aimed at quality of care is also described as advances, highlighting the scheduling of the first nursing consultation in primary care by the maternity ward, which facilitates access to healthcare services, the reception of the mother-baby dyad, the active search at home, multidisciplinary articulation and intersectorality^(9)^. These strategies have been highlighted for facilitating access and providing more efficient and humanized care, for ensuring continuity of care after hospital discharge, promoting healthy development, in addition to strengthening the bond between the family and healthcare services^(28)^.

Likewise, other actions considered advances in childcare include the implementation of child assessment instruments, such as the Child Health Booklet, the promotion of health education during consultations and the possibility of monitoring with specialized service programs, such as multidisciplinary teams in PHC^(26)^.

In relation to the COVID-19 pandemic, the country faced difficulties in monitoring childcare in primary care. Isolation measures and the risk of contagion made in-person consultations difficult. Therefore, there was a need to implement strategies that enable adequate care, such as telemonitoring, teleconsultation and the use of mobile applications^(29)^.

Even so, it is common to find in literature challenges similar to those described by study participants. The high demand for nursing professionals in healthcare services, the low adherence of users to participate in activities in the unit and absenteeism in appointments are factors that hinder the establishment of bonds^(22)^. Thus, the high demand for these professionals makes it difficult to provide the individualized attention needed to monitor child growth and development, limiting the time available for guidance and educational interventions for parents. As childcare requires a bond of trust between professionals and families, this ends up becoming challenging, as it can lead to discontinuity of care, lack of adherence to guidelines and even compromise of children's health and development.

In relation to absenteeism in appointments, it is also important to highlight the geographical difficulties in accessing the unit. Recent studies report that geographical distance is a factor that hinders access to healthcare services, since people are unable to reach the professionals^(30,31)^. Factors such as the remote location of the units, lack of transportation and even the socioeconomic conditions of families can result in non-attendance of appointments. This absence prevents the early detection of health problems and the monitoring of proposed interventions, thus creating a gap in preventive care.

Regarding the multidisciplinary team performance in childcare consultations, teamwork becomes essential for comprehensive care. However, despite childcare being shared between physicians and nurses, it is common to come across practices focused on the biomedical model and the treatment of diseases over health promotion and disease prevention^(31)^. Furthermore, monitoring of the child is generally done without taking into account the mother's perception of the child's growth and development, and health education actions end up being limited to breastfeeding and hygiene^(19)^.

Therefore, the need for professional development is highlighted, as mentioned by one of the participants. The lack of investment by educational institutions in training and professional qualification is an essential factor in promoting nurses' empowerment to monitor child growth and development in primary care^(26)^. This knowledge gap can result in inappropriate approaches, making it difficult to identify early health problems and promote preventive interventions. As a result, quality of care is affected, harming children's long-term development and health, and underestimating the importance of comprehensive and continuous care.

### Study limitations

The research has limitations related to the sample, which was composed of professionals from a FHS unit in each health district of the municipality studied, which makes it impossible to generalize the data.

It is worth noting that there were no discrepancies between the QUAN and Qual data. All nurses and managers brought in their narratives the facilities and difficulties encountered in carrying out nursing consultations in childcare, in addition to their postures and attitudes, which permeate the attention to this care in child healthcare today.

### Contributions to health, nursing or public policy

The study brought some advances and challenges in nursing consultation in childcare, showing the importance of the work of nurses in the area of child health, which leads to (re)thinking academic training and continuing education for child care performance within the scope of PHC.

## FINAL CONSIDERATIONS

Childcare is essential for promoting care and attention to child development, with nurses being the professional interlocutor for actions involving care and monitoring of children and their families in the Healthcare Network.

Managers value and recognize the relevance of nurses' work in childcare, but there are challenges that need to be overcome and discussed in multidisciplinary meetings of FHSs, in order to improve coverage in care for children and their families, aiming for comprehensiveness and longitudinality.

## Data Availability

The research data are available only upon request.

## References

[1] Ministério da Saúde (BR) (2018). Política Nacional de Atenação Integral à Saúde da Criança: orientações para implementação [Internet].

[2] Ministério da Saúde (BR) (2022). Caderno de Atenação Básica nÂ°33: Saúde da Criança: Crescimento e Desenvolvimento [Internet].

[3] Bugs CV, Monteiro AS, Ribeiro AC, Kleinubing RE, Disconsi FD, Schanne FF (2023). Facilitadores e barreiras da consulta de enfermagem em puericultura. Rev Eletron Acervo Saude..

[4] Coêlho AF, Nascimento NC, Santos MC, Ferreira MD, Carvalho DD, Almeida AG (2023). A importância da assistência de enfermagem no acompanhamento de puericultura: uma revisão integrativa. Braz J Dev..

[5] Reis KG, Serranegra NV, Varela AL, Almeida PA, Carrer MO, Barreto CP (2024). Consulta de enfermagem em saúde da criança e competências para Enfermeiros de Prática Avançada. Rev Esc Enferm USP.

[6] Yorinobu ACY, Andrade PR, Cruz AC, Silva L, Costa P, Belela-Anacleto ASC (2020). Contribuições do genograma e do ecomapa em consultas de enfermagem em puericultura. Rev Soc Bras Enferm Ped..

[7] Resende RA, Alflen PT, Silva VH, Soares VH, Gomes FA, Toniasso IM (2023). Saúde da criança: desafios da adesão à puericultura em uma Estratégia de Saúde da Família em Rondonópolis, Sul de Mato Grosso. Braz J Health Rev..

[8] Vieira DS, Brito PKH, Fernandes LTB, Reichert APS (2021). Consulta de enfermagem à criança na atenação primária à saúde: uma devolutiva de dados pesquisados. Rev Bras Enferm..

[9] Cavalheiro APG, Silva CL, Veríssimo MLOR (2021). Consulta de enfermagem à criança: atuação do enfermeiro na Atenação Primária à Saúde. Enferm Foco..

[10] Creswell JW (2010). Projeto de pesquisa: métodos qualitativo, quantitativo e misto.

[11] Tombolato MA, Santos MA (2020). Análise Fenomenológica Interpretativa (AFI): fundamentos básicos e aplicações em pesquisa. Rev Abord Gestalt..

[12] Hong QN, FÃbregues S, Bartlett G, Boardman F, Cargo M, Dagenais P (2018). The Mixed Methods Appraisal Tool (MMAT) version 2018 for information professionals and researchers. Educ Inf..

[13] Ministério da Saúde (BR) (2024). Caderneta da Criança: Menina : Passaporte da cidadania. 7Âª ediação [Internet].

[14] Bardin L (1977). Análise de conteúdo.

[15] Ministério da Saúde (BR) (2012). Política Nacional de Alimentação e Nutriação [Internet].

[16] Ministério da Saúde (BR) (2009). Matriz de ações de alimentação e nutriação na atenação básica de saúde [Internet].

[17] Soares AR, Guedes ATA, Vieira DS, Pedrosa RKB, Toso BRGO, Collet N (2022). Perception and use of the Child's Health Handbook by professionals and mothers: an interactionist approach. Rev Rene..

[18] Souza ND, Silva Pereira LP, Silva SV, Paula WKAS (2019). Vigilância e estímulo do crescimento e desenvolvimento infantil. Rev Enferm UFPE..

[19] Ministério da Saúde (BR), Secretaria de Ciência, Tecnologia, Inovação e Complexo da Saúde (2023). Protocolo Clínico e Diretrizes Terapêuticas Anemia por Deficiência de Ferro [Internet].

[20] Costa LS, Santos JD, Andrade GE, Silva AM, Borges RL, Sousa DC (2023). Análise dos casos de hipovitaminose A no brasil no intervalo de 2015 a 2022, com base nos dados do DATASUS. Braz J Implantol Health Sci..

[21] Biasucci G, Donini V, Cannalire G (2024). Rickets Types and Treatment with Vitamin D and Analogues. Nutrients..

[22] Santos ANS, Lopes CET, Mello ECA, Sousa CAM, Bezerra MAC, Silva LM (2024). Educação permanente e atenação básica na saúde: a importância do aprimoramento do conhecimento dos profissionais que trabalham na atenação básica na saúde para a demanda da população local em um município do Estado do Ceará. CLCS [Internet].

[23] Sociedade Brasileira de Pediatria (SBP) (2020). Os acidentes são evitáveis e na maioria das vezes, o perigo está dentro de casa![Internet].

[24] Almeida LA, Torres BVS, Silva JS, Silva RCM, Vieira ACS (2023). Prevenação de accidentes domésticos en la primera infancia: una revisião integradora. Rev Urug Enfermeria..

[25] Fundação Osvaldo Cruz (Fiocruz) (2022). Vacinação infantil sofre queda brusca no Brasil [Internet]. Fiocruz.

[26] Reis LM, Souza KD, Alcântara DS, Vieira MVP, Brito AKL (2024). Atuação do enfermeiro em puericultura. Rev Contemp..

[27] Bastos Ã‰P, Pinto LF (2024). Avaliação do vínculo longitudinal com o usuário e sua relação com a residência em medicina de família e comunidade em uma área do município do Rio de Janeiro, Brasil. Cienc Saude Colet..

[28] Sirimsi MM, Loof HD, Broeck KV, Vliegher KD, Pype P, Remmen R (2022). Scoping review to identify strategies and interventions improving interprofessional collaboration and integration in primary care. BMJ Open..

[29] Shibukawa BMC, Uema RTB, Piran CMG, Fonseca BS, Furtado MD, Merino MFGL (2022). Repercussions of the pandemic of COVID-19: care of the pediatric population in Primary Health Care. Rev Rene..

[30] Shibukawa BMC, Rissi GP, Uema RTB, Furtado MD, Merino MFGL, Higarasji IH (2023). Absenteísmo nos serviços de saúde da criança: uma revisão sistemática. Rev Bras Enferm..

[31] Vieira NFC, Machado MFAS, Nogueira PSF, Lopes KS, Vieira-Meyer APGF, Morais APP (2021). Factors influencing user satisfaction with Primary Health Care. Interface (Botucatu).

