# Supplementary material for: Nursing consultation in childcare in nurses' and managers' voices: a mixed methods study
Source: Rev Bras Enferm. 2025 Dec 8;78(4):e20240657. doi: 10.1590/0034-7167-2024-0657 (PMC12716679; doi:10.1590/0034-7167-2024-0657)
Supplement: Translation

# Translation: Consulta de enfermagem em puericultura nas vozes dos enfermeiros e gestores: estudo de métodos mistos

**DOI:** 10.1590/0034-7167-2024-0657pt

**Authors:** Amanda Teixeira Martins, Vanessa Faria de Freitas, Larissa Carvalho de Castro, Thayane Ingrid Xavier de Andrade, Wiara Viana Ferreira, Elaine Cristina Rodrigues Gesteira

**Affiliations:** I Universidade Federal de São João del-Rei. Divinópolis, Minas Gerais, Brasil; II Universidade Estadual de Campinas. Campinas, São Paulo, Brasil

**Keywords:** Saúde da Criança, Atenação Primária à Saúde, Assistência Integral à Saúde, Crescimento e Desenvolvimento, Cuidado da Criança

## Abstract

**Objetivos:**

investigar o processo de trabalho dos enfermeiros em relação à s consultas de enfermagem em puericultura.

**Métodos:**

pesquisa exploratória de abordagem mista, em que dados quantitativos foram coletados concomitantemente com dados qualitativos. Foram incluídos na pesquisa enfermeiros e gestores.

**Resultados:**

as entrevistas foram realizadas com nove enfermeiros e oito gestores de unidades da Estratégia Saúde da Família na região Centro-Oeste de Minas Gerais. Após a análise, emergiram quatro categorias temáticas: Orientações mais comuns durante as consultas de enfermagem em puericultura; Avanços da consulta de enfermagem em puericultura; Desafios da consulta de enfermagem em puericultura; e Protagonismo do enfermeiro nas consultas de puericultura.

**Considerações Finais:**

o enfermeiro é o profissional interlocutor das ações que envolvem o atendimento e seguimento infantil na Rede de Atenação à Saúde, mas há desafios que precisam ser superados, a fim de melhorar a cobertura no atendimento dessas crianças e suas famílias, almejando integralidade e longitudinalidade.

## INTRODUÇÃO

A primeira infância, período de 0 a 5 anos, constitui um momento decisivo para o desenvolvimento saudável do ser humano. Desde a década de 80, com o Programa de Assistência Integral à Saúde da Criança, há o desafio de estabelecer e executar políticas e programas que garantam as condições ideais para o pleno crescimento e desenvolvimento infantil com foco na integralidade do cuidado^(1)^.

De acordo com a Política Nacional de Atenação Integral à Saúde da Criança, as ações estratégicas para o atendimento universal e equitativo incluem: o acesso atualizado da Caderneta de Saúde da Criança; a qualificação dos profissionais de saúde para o atendimento das crianças e de suas famílias na Atenação Primária à Saúde (APS); a mobilização social e de comitê de especialistas para o Desenvolvimento Integral da Primeira Infância no Sistema Ãšnico de Saúde; e o apoio na implementação do Plano Nacional pela Primeira Infância^(1)^.

Nesse contexto, o acompanhamento do crescimento e desenvolvimento infantil deve ser realizado na APS por meio da Estratégia Saúde da Família (ESF), e ao enfermeiro cabe a atribuiação de realizar a consulta de enfermagem na puericultura, estabelecendo o vínculo entre o serviço e a comunidade, propiciando a comunicação e relação harmoniosa com a criança e sua família, além de realizar a sistematização da assistência, de modo a levantar os problemas de saúde e estabelecer um planejamento junto à família para a tomada de decisão, direcionando-as ao cuidado por meio da promoação, prevenação e reabilitação de agravos^(2-4)^.

Desse modo, a puericultura deve ser compartilhada entre médicos e enfermeiros, a fim de assegurar o seguimento da criança, conforme o que é preconizado pelo Ministério da Saúde, sendo sete consultas no primeiro ano de vida, duas, no segundo, e uma consulta anual, a partir dos dois anos de idade até completar nove anos^(5)^. Além da vigilância do crescimento e desenvolvimento infantil, a consulta de enfermagem é o momento oportuno para criar estratégias que coloquem os membros familiares como participantes ativos do cuidado à criança, incluindo o exercício da parentalidade, as relações com o ambiente interno e externo, bem como o estímulo para o crescimento e desenvolvimento harmonioso, que irão determinar a fase adulta, sendo o alicerce para o futuro com indivíduos saudáveis, humanitários e que sejam capazes de se relacionar e produzir, a fim de construir um ambiente favorável para as próximas gerações^(6)^.

No entanto, alguns estudos evidenciaram fragilidades na consulta de enfermagem em puericultura em diferentes estados do país como: a realização incompleta da consulta de enfermagem no que se refere à anamnese, exame físico, educação em saúde e acolhimento das famílias com as suas singularidades^(7,8)^; a falta de capacitação dos enfermeiros, consultas centradas na doença e não na prevenação, orientações restritas à amamentação e higiene^(5)^; e ainda barreiras inerentes à própria unidade de saúde, como a escassez de recursos humanos e materiais, entre outros que podem dificultar o acesso da família e da criança para uma efetiva puericultura^(9)^.

Diante do exposto, questiona-se: como os enfermeiros têm conduzido as consultas de enfermagem em puericultura? Quais as facilidades e dificuldades encontradas pelos enfermeiros e gestores para a execuação da puericultura?.

Acredita-se que, investigando as consultas de enfermagem em puericultura, haverá a identificação do processo de enfermagem executado e o levantamento das barreiras humanas e físicas que impedem a efetividade no seguimento para um cuidado integral das crianças, de forma a contribuir com propostas que visem à qualidade da assistência prestada.

## OBJETIVOS

Investigar o processo de trabalho dos enfermeiros em relação à s consultas de enfermagem em puericultura.

## MÉTODOS

### Aspectos éticos

Os preceitos éticos e legais referentes a pesquisas com seres humanos foram atendidos de acordo as Resoluções nº 466/2012 e nº 510/2016. O artigo foi aprovado pelo Comitê de Ética em Pesquisa da Universidade Federal de São João del-Rei, *Campus* Centro-Oeste, e Certificado de Apresentação e de Apreciação Ética. Todos os participantes assinaram o Termo de Consentimento Livre e Esclarecido.

### Tipo de estudo

Trata-se de pesquisa de métodos mistos, em que os dados quantitativos (QUAN) foram coletados concomitantemente com os dados qualitativos (Qual)^(10)^. O estudo QUAN foi do tipo exploratório, e a parte (Qual) foi descritiva, ancorada na fenomenologia hermenêutica, na qual a compreensão do vivido se dá pela interpretação dos pesquisadores^(11)^, porém ambos dados tiveram a mesma valorização de peso. A abordagem mista foi norteada pela ferramenta *Mixed Methods Appraisal Tool*, que foi desenvolvida para avaliar criticamente diferentes desenhos de estudo, identificando critérios metodológicos relevantes para avaliar a qualidade de estudos, como os estudos de métodos mistos^(12)^.

Optou-se por esta abordagem mista por permitir uma resposta abrangente e aprofundada sobre o fenômeno estudado, considerando os eixos norteadores que envolvem a consulta de enfermagem em puericultura na ótica quantitativa, e com os dados Qual, houve a oportunidade de compreender a experiência vivenciada. O uso de único método implicaria o conhecimento do fenômeno estudado, apenas de forma quantitativa, sobre o que os enfermeiros sabiam sobre a puericultura, enquanto a realização das entrevistas aprofundadas ofereceu a oportunidade de clarificar aos pesquisadores as ações realizadas durante a consulta e de entendê-la na ótica dos enfermeiros e gestores dos distritos de saúde selecionados.

Desse modo, o método misto permitiu a convergência dos dados, subsidiando a interpretação dos resultados, o que remeteu o conhecimento e a apreensão sobre as percepções e atitudes que envolvem a prática da puericultura realizada pelos enfermeiros na equipe multiprofissional, almejando destacar os avanços alcançados desta prática na atenação à saúde da criança e os desafios ainda existentes.

### Cenário do estudo

O estudo foi realizado em um município da região Centro-Oeste de Minas Gerais, que possui oito distritos sanitários: Central, Norte, Nordeste, Sudeste, Sul, Sudoeste, Oeste e Noroeste.

Dessa forma, foram incluídos na pesquisa os enfermeiros e gestores de uma ESF de cada região sanitária, elegíveis por conveniência, as quais eram campos de prática da graduação e da pós-graduação *lato sensu*, nível residência, de uma universidade pública da região Centro-Oeste mineira. Fez-se o contato prévio com os enfermeiros e gestores, por meio de ligação telefônica, nas respectivas unidades de saúde. Excluíram-se os profissionais que estiverem afastados por licença médica, licença sem vencimento e de férias.

### Coleta e organização dos dados

Os dados foram coletados de setembro de 2023 a junho de 2024. A amostra foi composta por nove enfermeiros e oito gestores. A coleta com os enfermeiros foi iniciada por meio de uma entrevista aberta baseada em perguntas sobre anamnese, exame físico, educação em saúde, condutas ao término das consultas e facilidades/desafios, a fim de conhecer e compreender as suas percepções acerca das consultas realizadas. Salienta-se que as entrevistas foram realizadas em locais privativos na unidade de saúde e após o consentimento dos participantes.

Ao término de cada entrevista com o enfermeiro, um questionário foi aplicado contendo dez questões de múltipla escolha abordando pontos específicos da consulta de enfermagem segundo a Caderneta de Saúde da Criança do Ministério da Saúde, a fim de investigar o processo de trabalho^(13)^. Contudo, as perguntas eram diferentes das realizadas na entrevista, com intuito de não influenciar as respostas e para complementar o estudo.

Para os gestores, foi aplicada uma entrevista aberta com duas perguntas centrais envolvendo a visão que tinham em relação à consulta realizada pelos enfermeiros, e os desafios e potencialidades existentes na área de abrangência de sua gestão.

Na etapa de coleta (Qual), as entrevistas foram gravadas e transcritas após o término. Os participantes foram identificados com as letras "E" (enfermeiro) e "G" (gestor), seguindo a sequência numérica de sua entrevista (E1, E2, E3, G1, G2, G3, sucessivamente). Os dados advindos das entrevistas passaram por três fases preconizadas pela análise de conteúdo^(14)^.

As três etapas foram pré-análise, exploração do material, codificação e classificação, e tratamento dos resultados obtidos e interpretação, com elaboração das categorias temáticas. Os códigos gerados foram analisados por três pesquisadoras de forma simultânea e independente.

Salienta-se que, para a realização das entrevistas e a aplicação dos questionários, duas pesquisadoras deste estudo foram as executoras, as quais fizeram o contato telefônico no centro de saúde de cada distrito selecionado, realizando o convite e o agendamento do dia e horário de preferência do enfermeiro e do respectivo gestor.

A equipe de coleta foi composta por uma pesquisadora enfermeira, mestre e doutora em enfermagem, e outras pesquisadoras são acadêmicas de enfermagem, sendo uma delas bolsista de iniciação científica, todas com experiência na conduação de entrevistas, além da experiência no atendimento de consultas de enfermagem em puericultura. Embora as unidades de ESF fossem campos de práticas da graduação e pós-graduação, buscou-se por conveniência profissionais não conhecidos pelas pesquisadoras, a fim de garantir o rigor metodológico.

### Análise dos dados

Para análise dos dados QUAN, os dados foram lançados e armazenados no *software Microsoft Office Excel*. Em seguida, foram analisadas em percentuais, de acordo com as respostas assinaladas à s perguntas, as quais foram descritas em um quadro apontando a alternativa que seria a correta, a resposta mais frequente respondida pelos enfermeiros e a porcentagem de acertos para cada pergunta realizada.

Ressalta-se que não foram aplicados testes de comparação ou associação para os resultados do questionário, pois a amostra foi limitante para realizar inferências estatísticas, contudo apresentam-se os resultados da estatística descritiva. A versão final do banco de dados foi checada por duas pesquisadoras.

A triangulação dos dados foi executada mediante o produto do material empírico advindo dos questionários e entrevistas, a fim de responder ao objetivo por meio da complementaridade de informações, o que permitiu uma visão mais ampla sobre o processo de enfermagem executado na puericultura.

## RESULTADOS

Os dados do estudo foram extraídos ao mesmo tempo com entrevistas e aplicação de questionários para os enfermeiros, sendo aplicadas as entrevistas em um primeiro momento, a fim de evitar viés, pois o questionário continha questões que poderiam influenciar as respostas. Contudo, a análise foi triangulada, permitindo identificar quatro categorias temáticas: Orientações mais comuns durante as consultas de enfermagem em puericultura; Avanços da consulta de enfermagem em puericultura; Desafios da consulta de enfermagem em puericultura; e Protagonismo do enfermeiro nas consultas de puericultura.

As condutas mais comuns durante a realização da puericultura estavam relacionadas, principalmente, ao aleitamento materno exclusivo/alimentação (100% de acerto no questionário), à suplementação e outras medicações em uso (66,6% de acerto), à s orientações para a prevenação de acidentes, à estimulação do desenvolvimento da criança (33,3%) e à s orientações para completude do calendário vacinal. Além disso, também se observou que alguns profissionais realizam o agendamento das próximas consultas de puericultura.

Destacaram-se como facilidades: a formação do vínculo com a família desde o acompanhamento no pré-natal; os instrumentos de avaliação da criança; a promoação da educação em saúde na consulta; a melhora dos indicadores de saúde; as possibilidades de acompanhamento com programas de serviço especializados; os avanços tecnológicos; a agenda programada de atendimento; e as ações e programas voltados para a atenação primária.

Em relação aos desafios, destacaram-se a necessidade de maior adesão dos pais e responsáveis à consulta de puericultura, as fragilidades na integração da equipe multiprofissional, a necessidade de atualização profissional, a alta demanda do enfermeiro no serviço de saúde e a localização da unidade, o que dificulta o acesso do usuário e limitações de integração com outros setores de atenação.

Ademais, os gestores sinalizaram sobre a importância da atuação do enfermeiro como membro essencial da equipe multiprofissional, detendo obrigações e compromissos relacionados à promoação, prevenação e manutenação da saúde infantil.

Os discursos dos enfermeiros e gestores foram analisados e apresentados de acordo com os códigos e categorias apresentados no Quadro 1.

**Quadro 1:** Categorias, códigos e discurso dos entrevistados, Divinópolis, Minas Gerais, Brasil, 2023.

Código	Categoria	Discursos dos enfermeiros e gestores
Aleitamento materno Alimentação complementar Medicações profiláticas Informação sobre prevenação de acidentes na infância Estímulo para o desenvolvimento da criança Identificação de atraso Vacinas Agendamento com outros profissionais	1 - Orientações mais comuns durante as consultas de enfermagem em puericultura	*Oriento os pais quanto à alimentação, quanto ao aleitamento, aleitamento materno exclusivo até os 6 meses. O aleitamento materno até os 2 anos de idade. Introduação de alimentação saudável* [...]. E2 [...] *eu oriento o cuidado, a importância, os benefícios do aleitamento, os vários alimentos, benefícios da amamentação exclusiva tanto para a criança quanto para a mãe, quanto também financeiramente que o leite de fórmula onera bastante* [...]. *Então, sempre esse cuidado com a alimentação de alimentos naturais, de continuar com a amamentação, mesmo com a introduação alimentar* [...]. E3 *Se ela tiver em aleitamento materno exclusivo, eu vou incentivar o aleitamento; vou parabenizar a mãe pelo aleitamento materno exclusivo; eu vou dar orientações de cuidados gerais com a criança de quando é necessário o uso de complemento de algum tipo de vitamina* [...]. E7 [...] *eu comento muito sobre a medicação que é diferente, do sulfato ferroso, principalmente, que a partir dos 6 meses, a gente orienta sobre a importância da medicação tanto também da vitamina D* [...]. E8 [...] *a prevenação de acidentes, no caso, a posiação para dormir, de preferência dormir no berço* [...]. *Quando ela começa a caminhar, engatinhar, cuidado com peças de brinquedo, pequena tampa de caneta, moedas, peças pequenas, ficar em casa, quina de móveis, gaveta de facas, a gaveta de utensílios cortantes é tomada, essas coisas, basicamente*. E3 [...] *se tiver algum atraso de movimento, posso pedir uma orientação para a fisioterapeuta da unidade. Ela ajuda muito nessa questão. Dependendo da idade, posso caminhar para o PIPA* [...]. E1 [...] *orienta de estimulação, ensino os exercícios que a mãe tem que fazer cada etapa por* [...] *pra tá estimulando o bebê pro desenvolvimento* [...]. E5 *Eu mostro todos os gráficos de desenvolvimento e do crescimento. Eu geralmente oriento como estimular o bebe de acordo com a idade* [...]. E9 [...] *faço o agendamento do retorno ou encaminhamento para algum ponto da rede ou para outro profissional quando necessário.* E4
Vínculo com a família Papel do enfermeiro Melhoria nos indicadores de saúde Acesso à s tecnologias de cuidado Acompanhamento de qualidade Puericultura compartilhada	2 - Avanços da consulta de enfermagem em puericultura	[...] *criar um vínculo com a família, e esse vínculo é criado, na verdade, desde o pré-natal, porque, eu, como enfermeiro, faço consulta de pré-natal. Então, esse vínculo aí é importante, porque aí a gente vai ter uma longitudinalidade da usuária* [...]. E1 [...] *a aderência da família é um facilitador muito grande.* E2 *Ás vezes, o pediatra, ele foca mais na questão clínica e esquece de dar as outras orientações. Então, tem muita mãe que busca a puericultura na unidade de saúde porque à s vezes a gente* [refere-se ao profissional de enfermagem] *conversa mais um pouco com ela, né, orienta mais* [...]. E7 *Melhoria dos indicadores, quando a gente pensa em questão vacinal, questão de SISVAN, que é vigilância nutricional, mas o que eu mais vejo é essa questão mesmo, principalmente de desenvolvimento. Quando a gente, o enfermeiro, capta alguma alteração, já é encaminhado para o Programa de Intervenação Precoce. A gente está dando uma assistência qualificada, quando vem e principalmente detectando o mais precocemente possível.* G1 *Avanços no sentido da tecnologia que facilita tanto o enfermeiro pesquisar, se atualizar, pra ele conversar com a mãe. Aplicativos que podem ser usados para poder tá acompanhando o desenvolvimento da criança, para saber se está adequado ou não com a idade* [...]. G2 *Mas um avanço que eu vejo também, assim, é na questão das agendas, ter as agendas reservadas pra puericultura.* G5 *Acho que o principal avanço é esse mesmo, da gente conseguir acompanhar quase que 100% dessas crianças. É raro o mês em que a gente não consegue acompanhar 100% das crianças. Todos já sabem da qualidade desse acompanhamento na unidade, então esse foi nosso maior avanço, nosso maior ganho até hoje* [...]. G7 [...] *os programas, de certa forma, eles já vêm pra poder suprir essas dificuldades que a gente teria enquanto base, que são as medicações. A gente tem aqui muito acesso à s vitaminas que antes a gente não tinha, né? Eu acho que o que mais avançou é esse olhar de que a puericultura. Ela faz parte de todas as especialidades, ela não pertence só ao enfermeiro, e ele não tem que fazer essas consultas sozinho* [...]. G8
Articulação da Rede de Atenação à Saúde Escassez quantitativa de profissionais Falta de conhecimento sobre a saúde da criança Falta de capacitações Sobrecarga do enfermeiro Falta de envolvimento dos pais Dificuldades de acesso à unidade (localização geográfica) Dificuldades de referenciar a criança para outros serviços da RAS.	3 - Desafios da consulta de enfermagem em puericultura	[...] *necessidade de uma equipe multiprofissional mais estruturada para ajudar a gente no acompanhamento, porque, muitas vezes, a gente se sente muito sozinho, né? Tem, à s vezes, o profissional, mas eles não vêm em quantidade suficiente na unidade. Ás vezes, é uma vez na semana, só meio horário. Eles não se encontram, a gente não consegue discutir os casos direito, é super fragmentado* [...]. E4 [...] *de dificuldade, eu acho que quando a gente não tem conhecimento, a falta de conhecimento de alguma coisa que tem, né?!* [...] *mas aqui não tem capacitação, não* [...] *eu nem sei quando foi a última vez que a gente teve alguma educação permanente em saúde da criança* [...]. *Hoje em dia, um enfermeiro só pra tudo na unidade, pra tudo, tudo, tudo mesmo, pra esse tanto de agenda, esse tanto de demanda espontânea, sempre deixar disponível pra isso, dificulta pra gente fazer um acompanhamento do jeito que precisa, sabe?* E5 *Acho que uma maior sensibilização por parte dos pais, né, da importância do acompanhamento de puericultura. Ás vezes, né, eu vejo, assim, que muitos não entendem ou não querem seguir as orientações dos enfermeiros ou que outro profissional de saúde passa. E eu acho que tem mais dificuldade em seguir algumas orientações em áreas menos favorecidas* [...]. G1 [...] *desafio também a agenda do profissional enfermeiro na unidade de saúde, diante de todas as outras funções que ele tem. É difícil ele separar uma agenda para a puericultura* [...] *a primeira coisa que faz é desmarcar a agenda de puericultura, porque acaba não sendo considerada de muita urgência, e acaba que isso vai descredenciando o serviço. Os pais não vão levar muito a sério*. G2 *Aqui o principal desafio que a gente tem é uma questão de logística* [...] *essa parte da comunidade que mora longe, ela não tem à ´nibus direto pra cá. Então, o principal desafio nosso não é técnico, não é de acompanhamento, é essa questão da logística da localização da unidade* [...]. G7 [...] *o que eu tenho muita ressalva é que quando a gente precisa de um terceiro setor, de um segundo setor, é aí que a gente começa a encontrar os desafios, se a gente encontra uma criança onde ela apresenta algumas limitações* [...] *para poder conduzir essa criança para uma assistência mais especializada* [...] *nós enquanto unidade, a gente encontra alguns obstáculos* [...]. G8
Reconhecimento do papel exercido pelo enfermeiro Desafios para a família no cuidado com as crianças Valorização da consulta de enfermagem	4 - Protagonismo do enfermeiro nas consultas de puericultura	[...] *o enfermeiro é um profissional capacitado e apto para realizar tal procedimento* [...]. G1 *O enfermeiro é um servidor que fica mais próximo da unidade. Ele tá lá todos os dias. É mais fácil para ele fazer esse acompanhamento da criança, então é importante o enfermeiro fazer esse acompanhamento seguindo as datas preconizadas para poder diagnosticar algum atraso e conseguir dar uma intervenação no tempo certo, dar as orientações para os pais ou responsáveis da criança da forma correta* [...] *é a pessoa que tem mais proximidade com o usuário*. G2 [...] *vejo que é um momento que muitas vezes a família tem até maior adesão do que talvez uma consulta pediátrica* [...]. G3 *Se a gente fosse centralizar as puericulturas, vamos dizer, assim, no médico ou mesmo que seja num pediatra principalmente. A gente não conseguiria atender, ficaria uma demanda bem defasada de atendimentos* [...]. G5 [...] *enfermeira na ponta, né? Ela é a que vai realmente acompanhar esse desenvolvimento que eu acho que é prioritário, mesmo esse atendimento da enfermagem* [...]. G6 [...] *elas têm um conhecimento técnico muito bom, avançado, é uma capacidade técnica enorme de poder acompanhar essas crianças com a puericultura e além de ter uma capacidade de abordagem com as responsáveis, os pais, em relação a isso* [...]. G7

Foi realizada a aplicação do questionário com os enfermeiros (nove) incluídos no estudo. Entre os temas que compõem a puericultura, explicitam-se, no Quadro 2, as questões realizadas e a identificação dos acertos e erros no questionário.

**Quadro 2:** Relação das questões do questionário, alternativa correta e resposta que os participantes mais selecionaram e porcentagem de acertos em cada questão (n= nove participantes), Divinópolis, Minas Gerais, Brasil, 2023.

Questão 01) Assinale a alternativa que corresponde com clareza à definiação da consulta de enfermagem em puericultura.		
Alternativa correta	Resposta mais frequente	Resposta correta (%)
e) Todas as alternativas estão corretas	e) Todas as alternativas estão corretas	88,90%
Questão 02) A consulta de enfermagem em puericultura é realizada até a idade de:		
Alternativa correta	Resposta mais frequente	Resposta correta (%)
b) Até os 9 anos, 11 meses e 29 dias	b) Até os 9 anos, 11 meses e 29 dias	44,40%
Questão 03) Você está realizando a consulta de enfermagem em puericultura de um recém-nascido prematuro. Considerando que o recém-nascido nasceu há dois meses atrás, com 29 semanas, assinale a alternativa correta da idade corrigida:		
Alternativa correta	Resposta mais frequente	Resposta correta (%)
c) 37 semanas	c) 37 semanas	77,70%
Questão 04) Em relação à suplementação da vitamina A, é incorreto afirmar que:		
Alternativa correta	Resposta mais frequente	Resposta correta (%)
b) Crianças de 1 a 3 anos de idade devem ser suplementadas	b) Crianças de 1 a 3 anos de idade devem ser suplementadas	44,40%
Questão 05) Quanto à administração da suplementação profilática de sulfato ferroso, é correto afirmar:		
Alternativa correta	Resposta mais frequente	Resposta correta (%)
e) Todas as alternativas estão corretas	a) Para recém-nascido a termo, de peso adequado para idade gestacional em aleitamento materno, a recomendação é de 1 mg/kg peso/dia a partir do 6o mês até o 24o mês de vida	22,20%
Questão 06) Em relação à alimentação complementar, é correto afirmar que:		
Alternativa correta	Resposta mais frequente	Resposta correta (%)
b) Crianças em aleitamento materno exclusivo, a partir dos 6 meses, devem iniciar a alimentação complementar e o consumo de água	b) Crianças em aleitamento materno exclusivo, a partir dos 6 meses, devem iniciar a alimentação complementar e o consumo de água	100%
Questão 07) Qual dos marcos do desenvolvimento não está presente aos 2 meses?		
Alternativa correta	Resposta mais frequente	Resposta correta (%)
a) Busca ativa de objetos	a) Busca ativa de objetos	33,30%
	b) Segura objetos	
Questão 08) Até que idade você deve verificar o perímetro cefálico e abdominal?		
Alternativa correta	Resposta mais frequente	Resposta correta (%)
c) Até 2 anos de idade	c) Até 2 anos de idade	66,70%
Questão 09) Qual idade é recomendada para iniciar a retirada de fraldas da criança?		
Alternativa correta	Resposta mais frequente	Resposta correta (%)
c) Entre 2 e 3 anos	d) Nenhuma das alternativas está correta	22,20%
Questão 10) Quantas consultas de puericultura a criança deverá realizar até os 2 anos de idade?		
Alternativa correta	Resposta mais frequente	Resposta correta (%)
c) Nove consultas	c) Nove consultas	66,70%

Nos itens definiação e objetivos da consulta, idade corrigida, e alimentação complementar, foram alcançados mais de 70% de acertos, seguidos do exame físico, com a verificação do limite de idade para aferiação do perímetro cefálico e abdominal (66,7%), e da quantidade de consultas que a criança deve realizar até os 2 anos de idade (66,7%). Em contrapartida, as questões que obtiveram menores índices de acertos estão relacionadas à suplementação de vitamina A (44,4%), até qual idade a puericultura deve ser realizada (44,4%), marcos do desenvolvimento (33,3%), suplementação profilática de sulfato ferroso (22,2%) e retirada de fraldas (22,2%).

## DISCUSSÃO

Em relação à s orientações mais comuns dadas pelos profissionais, destaca-se quantitativamente o incentivo à amamentação, ação que corrobora o resultado obtido pelo Estudo Nacional de Alimentação e Nutriação Infantil do Ministério da Saúde, que mostram aumento nos índices de aleitamento materno. Ao comparar os dados com inquéritos nacionais anteriores, com base em indicadores de amamentação propostos pela Organização Mundial da Saúde, todos os indicadores melhoraram no Brasil. Ocorreu o aumento de 15 vezes na prevalência de aleitamento materno exclusivo entre as crianças menores de quatro meses, e de 8,6 vezes, entre crianças menores de 6 meses^(15)^.

Todavia, é importante ressaltar que vários fatores podem influir no sucesso do aleitamento materno; entre eles, alguns estão relacionados à mãe, como as posturas e atitudes assumidas diante da situação do amamentar, e outros se referem a fatores socioculturais, demográficos, socioeconômicos e de assistência no pré e pós-natal^(16,17)^.

Nessa perspectiva, a atuação dos profissionais de saúde é de grande valia para iniciar corretamente o aleitamento materno, apoiando e estimulando as mães na superação dos prováveis problemas precoces recorrentes nesse processo através de orientações simples, mas atualizadas e transmitidas com simpatia, ética, paciência e humanização, para assegurar e encorajar a mãe de sua capacidade em amamentar, caso seja seu desejo^(15)^.

Além disso, o aleitamento materno exclusivo até o 6Â° mês, oferecido até os 2 anos de idade ou mais, bem como a diversidade da alimentação, são fatores determinantes para a oferta de diferentes nutrientes, contribuindo para a prevenação de deficiências nutricionais.

Outras orientações muito discutidas são o estímulo ao desenvolvimento infantil e o acompanhamento dos marcos do desenvolvimento. Sabe-se que a análise do crescimento e do desenvolvimento infantil deve ser feita de forma regular, de modo que seja possível a detecação precoce de alterações, viabilizando as devidas condutas em tempo hábil, com o objetivo de proporcionar à criança oportunidades para um desenvolvimento adequado durante toda a infância, contribuindo para que suas potencialidades sejam manifestadas de forma a refletir positivamente por toda a vida^(18)^.

No que se refere à suplementação de micronutrientes, prevalece o conhecimento do enfermeiro sobre o sulfato ferroso em detrimento dos demais micronutrientes. Pode-se afirmar que 77,8% dos entrevistados sabem sobre a correta indicação para o recém-nascido a termo, de peso adequado para idade gestacional em aleitamento materno, com a suplementação de ferro a 1 mg/kg peso/dia a partir do 6Â° mês até o 24º mês de vida^(19)^.

Em relação à vitamina A, 44,4% dos entrevistados afirmam que as crianças de 1 a 3 anos de idade não precisam ser suplementadas; essa medida foi justificada a partir da baixa incidência de hipovitaminoses relacionadas à vitamina A na região Sudeste do país. Em contrapartida, entre os anos de 2015 e 2022, foram registradas 169 internações por deficiência de vitamina A no Brasil, problema de saúde pública da região Nordeste há décadas, com destaque para o semiárido baiano, Rio Grande do Norte, Ceará e Recife, em decorrência da elevada prevalência de carência clínica em vários grupos infantis^(20)^.

Já sobre a vitamina D, apenas um dos enfermeiros mencionou a suplementação dessa vitamina. A deficiência vitamínica em crianças pode resultar em diversas complicações, como atraso no crescimento, deformidades e fragilidades ósseas, e aumento do risco de fraturas. Além disso, a vitamina D também desempenha um papel vital na regulação do sistema imunológico, e sua carência pode contribuir para uma maior suscetibilidade a infecções respiratórias e outras doenças. Estudos têm demonstrado associações entre a deficiência de vitamina D na infância e o desenvolvimento de doenças crônicas na vida adulta, como diabetes tipo 1 e doenças autoimunes^(21)^. Tal fato reforça a relevância do aconselhamento e intervenções da enfermagem relacionados à nutriação nas consultas de puericultura.

Ã‰ importante ressaltar que o aleitamento materno até 2 anos ou mais e, consequentemente, a alimentação da mãe, são fatores determinantes para a concentração de vitaminas na criança. Assim, é recomendado que as lactantes consumam diariamente uma alimentação saudável com níveis adequados para suprir suas necessidades diárias^(2)^.

No que diz respeito ao desfralde, apenas 22,2% dos participantes sabiam qual a idade recomendada para iniciar a retirada de fraldas, ou seja, entre 2 e 3 anos^(13)^. Assim sendo, é adequado que a qualificação profissional esteja conectada com os anseios e necessidades da comunidade^(22)^. Além disso, é essencial que os enfermeiros orientem os pais a perceberem os sinais da maturidade neural infantil e façam o processo de desfralde com muita compreensão e naturalidade^(13)^.

Quanto à prevenação de acidentes, somente um enfermeiro discorreu sobre o assunto no momento de orientações dadas aos cuidadores, embora, de acordo com a Sociedade Brasileira de Pediatria, os acidentes sejam a principal causa de morte de crianças de 1 a 14 anos de idade no país. Cerca de 90% desses acidentes podem ser evitados com medidas simples de prevenação^(23)^. Inúmeros acidentes acontecem dentro de casa, onde, a princípio, as crianças devem ter liberdade para circular, explorar e se desenvolver de forma saudável e segura^(24)^. Diante do exposto, é imprescindível que o assunto seja amplamente discutido durante as consultas de puericultura e que os responsáveis sejam alertados sobre situações de perigo relacionadas à s mudanças na capacidade cognitiva e locomotiva das crianças ao decorrer de seu desenvolvimento.

Da mesma forma, apenas um dos enfermeiros entrevistados mencionou orientações sobre a vacinação. Segundo o Fundo das Nações Unidas para a Infância, a baixa cobertura vacinal no país deixa a população infantil exposta a doenças que antes não eram mais uma preocupação, como o sarampo, que foi erradicado no país em 2016 e, em 2018, voltou para a lista de doenças no Brasil. Além do sarampo, outras doenças que correm o risco de voltar a acometer as crianças são a poliomielite, a meningite, a rubéola e a difteria^(25)^.

Nesse contexto, é nítida a necessidade de a atenação primária reforçar a capacitação dos enfermeiros para a consulta de puericultura, além de organizar ações de continuidade do cuidado e de acompanhamento não somente para o atendimento da demanda espontânea. Para tal, é preciso buscar ativamente as famílias a partir do pré-natal e integrar o cuidado com ações de prevenação e promoação da saúde^(26)^.

Em conformidade com os dados apresentados pelos enfermeiros e gestores, é possível notar inúmeros avanços na consulta de enfermagem em puericultura^(3)^. Ressalta-se a formação do vínculo com a família como um dos avanços apontados nas entrevistas, importante para a longitudinalidade da assistência, que é fortalecido a partir de uma relação de confiança e respeito, permitindo a integralidade do cuidado e o acompanhamento a longo prazo desses usuários^(27)^.

Além disso, a implementação de ações voltadas à qualidade da assistência também é descrita como avanços, destacando-se o agendamento da primeira consulta de enfermagem na atenação primária pela maternidade, o que facilita o acesso aos serviços de saúde, o acolhimento do binômio mãe-bebê, a busca ativa em à ¢mbito domiciliar, a articulação multiprofissional e a intersetorialidade^(9)^. Essas estratégias têm sido destacadas por facilitar o acesso e proporcionar um cuidado mais eficiente e humanizado, por garantir a continuidade do cuidado após a alta hospitalar, promovendo um desenvolvimento saudável, além de fortalecer o vínculo entre a família e os serviços de saúde^(28)^.

Do mesmo modo, outras ações consideradas avanços da puericultura incluem a implementação de instrumentos de avaliação da criança, como a Caderneta de Saúde da Criança, a promoação da educação em saúde na consulta e a possibilidade de acompanhamento com programas de serviço especializado, como as equipes multiprofissionais na APS^(26)^.

Em relação à pandemia de COVID-19, o país se deparou com dificuldades para o acompanhamento da puericultura na atenação primária. As medidas de isolamento e o risco de contágio dificultaram as consultas presenciais. Dessa forma, viu-se a necessidade de implementar estratégias que possibilitem a assistência adequada, como telemonitoramento, teleconsulta e a utilização de aplicativos móveis^(29)^.

Ainda assim, é comum encontrar na literatura desafios semelhantes aos descritos pelos participantes do estudo. A alta demanda dos profissionais de enfermagem nos serviços de saúde, a baixa adesão dos usuários para participar das atividades na unidade e o absenteísmo nos agendamentos são fatores dificultadores para o estabelecimento de vínculos^(22)^. Dessa forma, a alta demanda desses profissionais dificulta a atenação individualizada necessária para o acompanhamento do crescimento e desenvolvimento infantil, limitando o tempo disponível para orientações e intervenções educativas aos pais, e como a puericultura exige um vínculo de confiança entre os profissionais e as famílias, isso acaba se tornando desafiador, pois pode levar à descontinuidade do cuidado, à falta de adesão à s orientações e até ao comprometimento da saúde e do desenvolvimento das crianças.

No que tange ao absenteísmo nos agendamentos, também é importante destacar as dificuldades geográficas de acesso à unidade. Estudos recentes relatam que a distância geográfica corresponde a um fator dificultador ao acesso ao serviço de saúde, uma vez que não conseguem chegar até os profissionais^(30,31)^. Fatores como a localização distante das unidades, a falta de transporte e até as condições socioeconômicas das famílias podem resultar em não comparecimento à s consultas. Essa ausência impede a detecação precoce de problemas de saúde e o monitoramento das intervenções propostas, criando, assim, uma lacuna no cuidado preventivo.

Em relação à atuação da equipe multiprofissional na consulta de puericultura, o trabalho em equipe se torna essencial para a integralidade do cuidado. No entanto, apesar de a puericultura ser compartilhada entre médicos e enfermeiros, é comum se deparar com práticas centradas no modelo biomédico e no tratamento de doenças em detrimento da promoação da saúde e prevenação de agravos^(31)^. Ademais, o acompanhamento da criança geralmente é feito sem levar em consideração a percepação da mãe sobre o crescimento e o desenvolvimento da criança, e as ações de educação em saúde acabam se limitando apenas à amamentação e à higiene^(19)^.

Sendo assim, destaca-se a necessidade de atualização profissional, como mencionado por um dos participantes. A falta de investimento das instituições de ensino na formação e na qualificação profissional é fator essencial para promoação do empoderamento do enfermeiro para a vigilância do crescimento e desenvolvimento infantil na atenação primária^(26)^. Essa lacuna de conhecimento pode resultar em abordagens inadequadas, dificultando a identificação de problemas de saúde precoces e a promoação de intervenções preventivas. Como consequência, a qualidade do atendimento é afetada, prejudicando o desenvolvimento e a saúde da criança a longo prazo, e subestimando a importância do cuidado integral e contínuo.

### Limitações do estudo

A pesquisa possui limitações relacionadas à amostra, a qual foi composta por profissionais de uma unidade de ESF de cada distrito sanitário do município estudado, o que impossibilita a generalização dos dados.

Ressalta-se que não houve divergências entre os dados QUAN e Qual. Todos os enfermeiros e gestores trouxeram em suas narrativas as facilidades e dificuldades encontradas para a realização das consultas de enfermagem em puericultura, além de suas posturas e atitudes, que permeiam a atenação deste cuidado na atenação à saúde da criança na atualidade.

### Contribuições para as áreas da saúde, enfermagem ou políticas públicas

O estudo trouxe alguns avanços e desafios da consulta de enfermagem em puericultura, mostrando a importância do trabalho dos enfermeiros na área da saúde da criança, o que remete (re)pensar a formação acadêmica e a educação continuada para a atuação do cuidado infantil no à ¢mbito da APS.

## CONSIDERAÇÕES FINAIS

A puericultura é essencial para a promoação do cuidado e atenação ao desenvolvimento infantil, sendo o enfermeiro o profissional interlocutor das ações que envolvem o atendimento e o seguimento das crianças e de suas famílias na Rede de Atenação à Saúde.

Os gestores valorizam e reconhecem a relevância do trabalho do enfermeiro na puericultura, mas há desafios que precisam ser superados e discutidos nas reuniões multiprofissionais das ESFs, a fim de melhorar a cobertura no atendimento à s crianças e suas famílias, almejando a integralidade e a longitudinalidade.

## Data Availability

Os dados de pesquisa só estão disponíveis mediante solicitação.
